# Testicular tissue re-implantation and the ‘hostile testis’

**DOI:** 10.1093/humrep/dead258

**Published:** 2023-12-22

**Authors:** Rod T. Mitchell, Jonathan Ives

**Affiliations:** 1 Centre for Reproductive Health, Institute for Regeneration and Repair, The University of Edinburgh, Edinburgh, UK; 2 Department of Paediatric Endocrinology, Royal Hospital for Children and Young People, Edinburgh, UK; 3 Centre for Ethics in Medicine, University of Bristol, Bristol, United Kingdom of Great Britain and Northern Ireland

## Testicular transplantation to restore fertility in males – who is it for?

Fertility Preservation in young males is a well-recognised area of clinical need. Currently, for males who are unable to produce viable sperm there are no proven clinical options to father their own biological child (Mulder et al, 2021). Cryopreservation of testicular tissue obtained following biopsy is increasingly being used to preserve spermatogonial stem cells (SSCs) within their testicular niche in prepubertal boys facing gonadotoxic treatments in the hope that the tissue can be used to generate sperm after they have completed their treatment (Goossens et al, 2020). This could involve re-transplantation of frozen-thawed testicular tissue back to the patient after they have reached adulthood. The field received a significant breakthrough in 2019 with the birth of Grady, the first non-human primate to be born using sperm derived from testicular tissue that had been harvested from a prepubertal Rhesus monkey prior to sterilising chemotherapy. Re-implantated testicular tissue was able to generate sperm that could be used for intracytoplasmic sperm injection (ICSI), resulting in the birth of a healthy baby monkey (Fayomi et al, 2019). However, transplantation of testicular tissue has yet to be shown to result in the generation of functional sperm in human. For such an approach to be successful in humans it is a prerequisite that the material to be transplanted contains viable SSCs that can undergo differentiation into functional sperm capable of generating offspring.

In this issue of Human Reproduction, Jensen et al (Jensen et al, 2023) report their findings from autologous transplantation of human testicular tissue in an infertile adult male. The study represents an advance for the field by providing evidence that the transplanted tissue can survive. However, the failure to generate sperm in the transplanted tissue raises questions regarding which patients might be considered suitable for this approach and for whom it may be unsuitable. The study also highlights the importance of publishing results from clinical trials reporting essentially negative findings regarding the original hypothesis to inform future clinical application of this approach.

## Re-implantation of testicular tissue - the ‘hostile testis’ hypothesis

For men with azoospermia, surgical sperm extraction is an established method to try to obtain sperm directly from the testis for assisted reproduction using intracytoplasmic sperm injection (ICSI). If no sperm are found it is not currently possible to generate a biological child and alternative methods of fatherhood, such as IVF using donor sperm or adoption, can be considered. In the reported case of an adult man with azoospermia, sperm were already found to be present in the testis following surgical testicular sperm extraction (TESE) and these could be extracted for ICSI. The sperm were able fertilise an egg but did not result in a viable pregnancy. The authors postulated that this is due to sperm being part of an undefined ‘hostile’ testicular environment and that removing, cryopreserving and re-transplanting selected areas of testicular material in an ectopic location would be able to sustain sperm production. In order for this to be of benefit to the patient, these sperm would need to be capable of fertilising an egg and have an improved likelihood of generating a viable pregnancy than sperm already present in the testis.

Whilst an intriguing concept, evidence to support a hypothesis that the ‘hostile’ environment affects the ability of sperm to result in a viable pregnancy or produce offspring is lacking. The hypothesis also relies on effective methods to remove the factors that contribute to the proposed ‘hostile’ environment. The authors attempt to do this by isolating apparently ‘normal’ tubules from the cryopreserved material prior to transplant. Unfortunately, no sperm were identified in the transplanted material and therefore it is not possible to conclude whether re-implantation of adult human testicular tissue can support sperm production. Questions about the presence of a ‘hostile testis’, what cellular or secretory factors would contribute to this, and how these factors can be effectively eliminated to generate sperm with an improved capacity to produce a viable pregnancy and offspring, compared with those obtained by TESE from the testis itself, remain to be answered. Conversely, an unidentified genetic cause for the severe spermatogenic defect would be unlikely to be improved.

## First in human studies – an ethics perspective

Notwithstanding the advances reported by Jensen et al, there remains an overarching question over when ‘first in human’ clinical procedures should be attempted, and under what conditions such procedures would be ethically acceptable.

First, as the authors describe in the paper, the underlying (possibly genetic) spermatogenic failure is the most likely reason why this procedure was not successful in improving the outcome for this particular patient. Admittedly, it is easy to say this with hindsight, but there were certainly reasons, available in advance, to suggest that the procedure was unlikely to work. When trying a procedure for the first time, it would be preferable to undertake it with a candidate on whom it may be most likely to work, to show proof-of-concept before attempting this in those with less likelihood of success.

Arguably, the approach taken might be justified if it can be framed within a narrative of ‘compassionate use’, in which untested interventions might legitimately be used when it is a person’s last – and only – hope of remedy. This is usually used in cases of potentially life-saving interventions (Raus et al, 2016); but, where the risks are proportionately low we might use the principle to justify surgery to make reproduction possible. Of course, this kind of attempt requires that the patient is fully informed of all the risks as well as potential benefits. Whilst the approach itself may be considered relatively low risk, there remains the possibility of physical complications from multiple surgical procedures and negative psychological impacts if unsuccessful. The plethora of widely used but unproven approaches to improve conception rates indicates a patients willingness to explore any avenue that might offer hope for success (Lundin et al, 2023).

It can be argued, of course, that an informed, capacitous person is permitted to expose themselves to those risks, however small the chance of success may be. This is true to some extent. What complicates it, however, is the opportunity cost, both in terms of the literal cost of the procedure, and the cost of the negative result – which we will now briefly discuss.

## The benefits of publishing negative results

Whilst it is certainly not common to do so, there is a widely recognised imperative to publish and make available negative results (Nimpf et al, 2020; Brassington et al, 2017). Though not routinely allocated space in journals, negative results are important because they can help prevent multiple groups from trying, and failing to do, the same things. This serves to preserve resource, protect patients and, additionally, reminds us that not all scientific enquiry results in glittering world changing findings; and that hypothesis testing often ends in a negative result which, far from being indicative of failure, usefully informs us of certain avenues that should not be pursued further. Accordingly, we applaud Jensen et al for seeking to publish, and the journal for publishing, this negative result with respect to the initial hypothesis.

However laudable publication may be, this paper does starkly highlight how delicate the balance sometimes is – especially when there are legitimate questions over the feasibility of the attempt. Given the concerns outlined above about the chances of success of the procedure with this patient, trying it out – and showing it to be unsuccessful – has potential to do a disservice to the science; as it might suggest to others that this procedure does not work when, in fact, it may require to be tested in a more suitable patient group. As such, it is important to read negative results as critically as one would read positive results and ensure that the baby is not thrown out with the proverbial bathwater – as we shall now consider.

## Boys facing gonadotoxic therapy – where does this leave us?

In addition to the existing impaired spermatogenesis, failure to generate sperm in this patient may be due to the re-implanted testicular tissue being obtained in adulthood. Degeneration of tissue and failure to generate sperm has been shown in studies involving transplantation of human adult testicular tissue in xenograft models (Geens et al, 2006; Schlatt et al, 2006). Therefore, this should not be taken as a sign that this approach will not benefit other patient groups. Indeed, prepubertal boys facing highly-gonadotoxic therapies are likely to represent the ideal patient group for future attempts at re-implantation of frozen-thawed testicular tissue, as part of a clinical trial. The key difference between these two patient groups is that in these boys the testicular tissues obtained prior to chemotherapy or radiotherapy will often be normal healthy testicular tissue with the potential for generating sperm that can be used to father children. This, coupled with the fact that the planned treatment is likely to remove any possibility of spermatogenesis in the exposed testis, makes these patients good candidates for testicular tissue re-implantation and contrasts with the situation in adult men with pre-existing severe abnormalities in spermatogenesis. Testicular biopsy for tissue cryopreservation has now been performed on thousands of boys worldwide and the expectation is that in many of these patients, re-implantation of testicular tissue would be the preferred option to generate sperm to allow them to have their own biological child. The birth of baby Grady has delivered the proof-of-principle that re-implantation of testicular tissue in adulthood is feasible for prepubertal boys facing gonadotoxic therapies and we await the first reports of functional sperm production in this patient group with great anticipation.
